# Beyond DNA barcoding: The unrealized potential of genome skim data in sample identification

**DOI:** 10.1111/mec.15507

**Published:** 2020-06-29

**Authors:** Kristine Bohmann, Siavash Mirarab, Vineet Bafna, M. Thomas P. Gilbert

**Affiliations:** ^1^ Section for Evolutionary Genomics The GLOBE Institute University of Copenhagen Copenhagen Denmark; ^2^ Department of Electrical and Computer Engineering University of California San Diego CA USA; ^3^ Department of Computer Science and Engineering University of California San Diego CA USA; ^4^ Center for Evolutionary Hologenomics The GLOBE Institute University of Copenhagen Copenhagen Denmark; ^5^ NTNU University Museum Trondheim Norway

**Keywords:** biodiversity, DNA barcoding, DNA reference databases, environmental DNA, K‐mers, next‐generation sequencing

## Abstract

Genetic tools are increasingly used to identify and discriminate between species. One key transition in this process was the recognition of the potential of the ca 658bp fragment of the organelle cytochrome c oxidase I (COI) as a barcode region, which revolutionized animal bioidentification and lead, among others, to the instigation of the Barcode of Life Database (BOLD), containing currently barcodes from >7.9 million specimens. Following this discovery, suggestions for other organellar regions and markers, and the primers with which to amplify them, have been continuously proposed. Most recently, the field has taken the leap from PCR‐based generation of DNA references into shotgun sequencing‐based “genome skimming” alternatives, with the ultimate goal of assembling organellar reference genomes. Unfortunately, in genome skimming approaches, much of the nuclear genome (as much as 99% of the sequence data) is discarded, which is not only wasteful, but can also limit the power of discrimination at, or below, the species level. Here, we advocate that the full shotgun sequence data can be used to assign an identity (that we term for convenience its “DNA‐mark”) for both voucher and query samples, without requiring any computationally intensive pretreatment (e.g. assembly) of reads. We argue that if reference databases are populated with such “DNA‐marks,” it will enable future DNA‐based taxonomic identification to complement, or even replace PCR of barcodes with genome skimming, and we discuss how such methodology ultimately could enable identification to population, or even individual, level.

## 1 FROM DNA BARCODING TO DNA MARKING

DNA sequences are increasingly being applied as a tool with which to assign identity to query samples, most famously through the use of so‐called “DNA barcodes” (Hebert, Cywinska, Ball, & deWaard, 2003). Originally conceived as a ca 658bp fragment of the organelle cytochrome c oxidase I (COI) gene to serve as a taxonomic tool for use in animal bioidentification, the idea was elegant. Users would PCR amplify and then Sanger sequence this marker, chosen based on their observations using lepidopterans as a test, to be conserved enough to be targeted with generic (pan‐taxa) primer sets, while variable enough to provide variation at the interspecific level (while similarly not varying at the intraspecific level). This elegant idea, of a barcoding region with which to tell species across life forms apart, quickly caught on, and subsequently a flurry of other organellar regions and markers and associated primer sets were proposed. For example, PCR amplification and sequencing of bacterial 16s rRNA as a tool for species identification was demonstrated shortly after the invention of PCR itself (Böttger, 1989; Wilson, Blitchington, & Greene, 1990), then introduced soon after to animals, including mammals (Taylor, 1996), amphibians (Vences, Thomas, van der Meijden, Chiari, & Vieites, 2005) and insects (Clarke, Soubrier, Weyrich, & Cooper, 2014). 12s was proposed for vertebrates (Riaz et al., 2011); Matk (Lahaye et al., 2008) and rbcl (Fazekas et al., 2008) for plants; ITS for fungi (Schoch et al., 2012) and so on.

As DNA barcoding's potential became increasingly apparent, it spurred rapid development in a range of associated laboratory and computational techniques to help optimize its performance, through facilitating efficient generation of high quality and economical data. In the laboratory, progress has principally been focused on decreasing the costs for generating single DNA reference and query barcodes—a key step for democratizing its use. For example, the state of the art is to use Illumina (Liu, Yang, Zhou, & Zhou, 2017) or PacBio (Hebert et al., 2018) technology to simultaneously sequence multiplexed amplicons derived from voucher specimens, so as to generate tens of thousands of sequences in parallel, thus decreasing sequencing costs to only a few cents per barcode (Hebert et al., 2018). A second avenue of progress relates to the development of computational methods designed to optimize the information potential of barcode data, in particular in light of challenges such as error within query barcode sequences, or incomplete or even erroneous reference databases (e.g. Bridge, Roberts, Spooner, & Panchal, 2003; Briski, Ghabooli, Bailey, & MacIsaac, 2016)). However, perhaps the most important of these developments was the realization that the power of barcoding is constrained by the quality of reference data against which to compare query sequences, thus the need for comprehensive and curated barcode reference databases based on the sequencing of vouchered information. Hebert and team's BOLD (Ratnasingham & Hebert, 2007) epitomizes this ideal, containing barcode sequences from over >7.9 million specimens (http://www.boldsystems.org/index.php, retrieved February 2020).

Recently, DNA reference databases are increasingly being complemented by shotgun sequencing‐based “genome skimming” alternatives (Bock, Kane, Ebert, & Rieseberg, 2014; Coissac, Hollingsworth, Lavergne, & Taberlet, 2016; Dodsworth, 2015; Marcus, 2018; Nevill et al., 2020; Zeng et al., 2018). In such approaches, while the original barcode loci are sequenced (Liu et al., 2013) with probability depending upon the coverage, the biggest benefits come from the sequencing and assembly of organellar genomes (Gillett et al., 2014) and through offering the potential to mine repetitive elements (such as nuclear rRNA repeats) out of the nuclear genome (Dodsworth et al., 2015; Dodsworth, Chase, Särkinen, Knapp, & Leitch, 2016; Krehenwinkel et al., 2019; Marcus et al., 2018; Turner, Paun, Munzinger, Chase, & Samuel, 2016). Unfortunately, much of the nuclear genome (as much as 99% of the sequence) is discarded. Ultimately, this can limit the power of discrimination at or below the species level (Rubinoff & Holland, 2005).

As such, we build on the suggestion first outlined by Coissac and colleagues (Coissac et al., 2016), and advocate that the full shotgun sequence data generated from voucher specimens could also be used to assign an identity (that we term for convenience here its *“DNA‐mark”*), without requiring any computationally intensive pretreatment (e.g. assembly) of reads. With such reference information in place, we argue that future studies that aim to assign an identity to query samples could complement, or even replace PCR of barcodes with shotgun sequencing, yielding data that could be matched to information in the reference database using computational methods that treat both the query and reference samples as “bags of reads” (Sarmashghi, Bohmann, Gilbert, Bafna, & Mirarab, 2019). We believe that this methodology ultimately could enable identification to population, or even individual, level.

## 2 THE LIMITS OF TRADITIONAL BARCODING

It is impossible to overstate the impact that traditional single‐locus DNA barcoding has had over the past 15 years, and it will without doubt continue to represent a fundamental pillar of many future studies. However, after such extensive use, its limitations are also now apparent, raising the obvious question as to whether these can be overcome? Principal among them is the taxonomic resolution at which traditional barcodes can effectively operate—having been chosen with the aim of discriminating at the species level (although even this is not guaranteed), they work suboptimally as one moves both below the species to other units that may interest end users—such as the population, or even individual, as well as up in taxonomic rank to Families, Orders, etc. due to sequence saturation making the resolution of deep lineage divergences very difficult (Chambers & Hebert, 2016; Marcus et al., 2018). This first of these problems is confounded by the “barcoding gap” challenge, namely that the genetic distance between taxonomic units is not a constant; thus while traditional barcodes may be effective in discriminating between different species in one genus, they may fail to perform on other genera (Shearer & Coffroth, 2008; e.g. Wiemers & Fiedler, 2007). A third limitation inherent to their relatively short length and their association with organelles, is they rarely can be used to resolve phylogenies with high statistical support, and their signal can be confounded due to phenomena such as hybridization, lateral transfer of organelles, and introgression (Duvernell & Aspinwall, 1995; Good et al., 2008; Marcus et al., 2018). Two further challenges relate to the DNA itself. The first of these relates to the minimum length of intact DNA templates required to successfully PCR amplify a barcode locus. The DNA content of many specimens of interest is often heavily degraded due to age, storage conditions or chemical treatment, and remaining fragments may simply be too short to allow initial PCR amplification step (Orlando, Gilbert, & Willerslev, 2015). And the last is that heavily degraded samples may also be contaminated with exogenous sources of DNA, which given the sensitivity of PCR, can potentially lead to the co (or even preferential)‐amplification of the contaminant over the true target (Hofreiter, Serre, Poinar, Kuch, & Pääbo, 2001).

**FIGURE 1:**
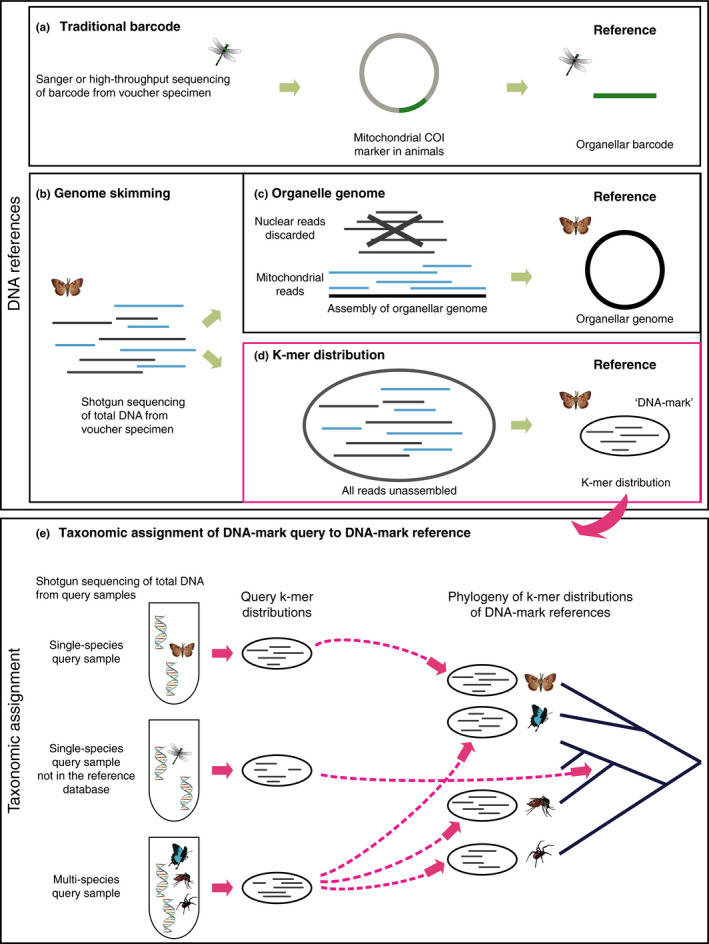
Methods to assign a genetic identity to voucher and query samples. (a) Traditional approaches are based on PCR amplification of barcode loci. (b) Increasingly genome skimming is used to bioinformatically mine the (c) barcode loci or whole organellar genomes from shotgun sequenced data. (d) We advocate that the remaining data could be used to assign a k‐mer profile to the specimen, (e) ultimately enhancing the resolution to which it can be identified (e)

The decreasing cost of sequencing using so‐called next‐generation sequencing (NGS) technologies has provided partial solutions to this problem, in particular thanks to the introduction of “genome skimming” approaches (Coissac et al., 2016). In their current implementation, DNA extracted from voucher specimens is converted into NGS libraries, shotgun sequenced to relatively low genome coverage, then either original barcode loci such as COI (Liu et al., 2013), or full organellar genomes, are reconstructed bioinformatically from this data (Figure 1) (Gillett et al., 2014). Thanks to library indexing, many samples can be multiplexed before sequencing, meaning that many tens (or even hundreds) of organellar genomes can be sequenced on a single sequencing run (even more, if coupled to target‐enrichment (Liu et al., 2016)). This yields a significant increase in information potential. This is further increased by the reduction in DNA preservation requirements when bypassing the conventional PCR step. For genome skimming, DNA fragments as short as 25–30 bp are usable, in stark contrast to the ca 700 bp requirement in traditional barcoding, which can hinder generation of reference sequences from old or badly preserved specimens. In light of these benefits, today several projects have actively chosen to employ genome skimming over traditional PCR to generate barcode‐like data, for example the PhyloAlps (phyloalps.org), NORBOL (norbol.org) (Alsos et al., 2020) and DNAmark (dnamark.ku.dk) initiatives, and in doing so are extending the concept of traditional DNA barcode reference databases (Hebert et al., 2003) to encompass organellar genome data. However, while this represents a natural development to traditional barcoding, we highlight that even this approach has its limits. Should sufficient genetic diversity and population structure exist in the target species, organellar genomes might enable us to narrow identification to the subspecies or even population level; however, unless organelle haplotypes are unique to individual organisms, their resolution stops here. Furthermore, inferences based on single nonrecombining loci (no matter how long) are notoriously susceptible to challenges such as incomplete lineage sorting, thus making them suboptimal for assigning identity or inferring evolutionary histories (Funk & Omland, 2003; McKay & Zink, 2010). Lastly and importantly, utilizing genome skimming with the sole intention of recovering organellar sequences simply seems wasteful, as it only exploits a fraction of the generated sequence data (although of course it is the norm for the full sequence data generated to be deposited in public databases, thus rendering them available for use in other studies). The nuclear DNA component of shotgun sequenced DNA extracts can represent > 99% of the reads (Liu et al., 2016), and we argue this holds valuable information that can further the goals of sample identification.

## 3 EXPLOITING THE POWER OF THE NUCLEAR GENOME

Given that the nuclear genome sequence of any nonclonal organism is a representation of its evolutionary history, it represents the ultimate source of information for those wishing to assign identity to samples. In theory, with enough reference data one could identify every genetically distinct organism on the planet. As such, if one looks to the future, the obvious desirable end goal would be to generate fully assembled nuclear genomes from both query and voucher samples and to do this across the entire Tree of Life, as advocated, for example, by initiatives such as the Earth BioGenome Project (Lewin et al., 2018) (https://www.earthbiogenome.org/), which are starting to be realized through projects such as the Darwin Tree of Life Project (https://www.sanger.ac.uk/science/collaboration/darwin‐tree‐life‐project). Unfortunately however, while sequencing technology is advancing at a remarkable rate thanks to the increases in accuracy, read length and overall output of platforms such as the PacBio Sequel II, which has allowed generation of largely complete genome assemblies for many organisms, the assembled nuclear genomes come with their own challenges. First, nuclear genomes are expensive to generate as they require sequencing to high depths of coverage. Second, the assembly is constrained by depth of sequencing and the repeat structure of the genome. On the one hand, if the depth of sequencing is high, then the computational power needed for the assembly is very high. On the other hand, sequencing depth cannot be too small either, as this will be problematic for successful assembly. Typically, a minimum depth of coverage is required that falls in the range of at least 50x for a relatively straightforward diploid organism (Sohn & Nam, 2018). A further challenge is repeat sequences, which when longer than the reads sequenced, can prevent unambiguous assembly. Repeats can be resolved by construction of mate‐pair/large insert libraries for short‐read technologies, and/or extraction of high molecular weight DNA and long‐read sequencing using single molecule sequencing. This in turn limits both which specimens can be used, and complicates the requisite laboratory equipment and skills.

In summary, the costs of assembling nuclear genomes are high, both with regards to the data generation and the computational assembly. This puts nuclear genomes well beyond the budgets and capabilities of most people actively interested in using DNA as a tool for routine taxonomic assignment of many samples. However, given that nuclear genome sequences are unique, regardless of whether they have been assembled into contigs, scaffolds or chromosomes, it follows that even unassembled shotgun sequence data might hold information that could be exploited for taxonomic assignment. And thus given such data are already being generated by current reference database genome skimming and genome projects, we argue that now is the time to explore its potential and develop suitable laboratory and computational tools for its exploitation.

## 4 UNLEASHING THE FULL POTENTIAL OF GENOME SKIMMING USING ASSEMBLY‐FREE METHODS

**FIGURE 2:**
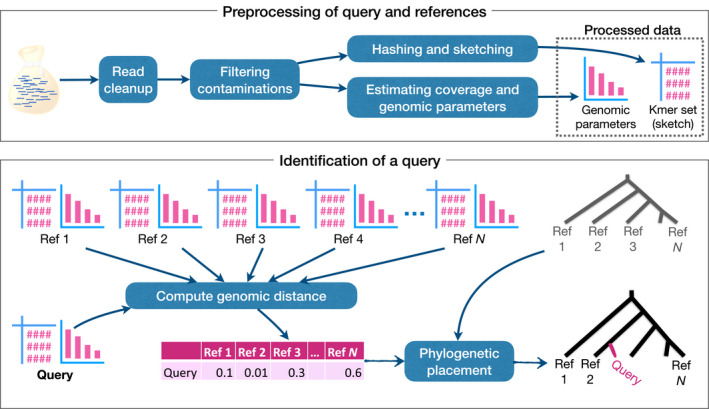
Overview of the DNA‐mark pipeline. Computational steps are shown in blue boxes, and one example tool that can be used in each step is shown below each box. For each set of reads (whether representing the voucher or the query), the sample has to be first preprocessed in several stages. First, reads are cleaned up to remove adapters, deduplicate reads and merge paired‐end reads. Then, extragenic reads need to be filtered out, typically by matching each read against a database of potential contaminants. The remaining reads need to be represented as k‐mers; the set of k‐mers need to be hashed and sketched for efficient storage and fast processing. Also, the coverage of the genome skim and properties of the underlying genome (e.g. its size and repeat structure) need to be estimated. Thus, the preprocessing (which needs to happen only once) generates both the k‐mer set and the genomic parameters, which are sufficient for sample identification. To identify a new query sample, we need to first compute its distance to the set of reference genome skims. The query can be assigned to the reference with the smallest distance. Alternatively, the query can be placed on a reference phylogenetic tree (which can be computed from the genome skims or can be retrieved from any other source)

How might we best exploit this residual nuclear DNA data? The ideal solution would be an approach that is fast, simple and efficient, and at least in the short term while sequencing costs are still in the range of >10 USD per GB (Rachtman, Balaban, Bafna, & Mirarab, 2020), restricts sequencing effort to a minimum. Our proposed solution is to use the unassembled reads from the nuclear genome (so‐called “bags of reads”) to perform the function currently assigned to barcodes (or organellar genomes), namely populate reference databases against which queries can be matched (Figures 1 and 2). Critically, such a method would need to be simple and intuitive, and computationally efficient—both with regard to data processing and storage.

Coissac et al. (2016) have suggested that assembly‐free and mapping‐free methods (Blaisdell, 1986; Fan, Ives, Surget‐Groba, & Cannon, 2015; Maillet, Collet, Vannier, Lavenier, & Peterlongo, 2014; Song et al., 2013; Vinga & Almeida, 2003) naturally meet many of these criteria. They are typically fast and conceptually simple. Following this aim, several groups have recently developed methods specifically aimed at handling characteristics specific to genome skimming, including low coverage and sequencing errors (Sarmashghi et al., 2019; Tang, Ren, & Sun, 2019). Indeed, many alignment‐free methods are available and their application to genome skims should be explored. We note that accurate analyses of skimming data will require several computational components (Figure 2). In recent years, a new toolkit of methods for analysing skimming data has started to emerge. Below, we discuss some of these advances, focusing specifically on analyses based on short oligomers, or k‐mers.

### 4.1 K‐mer‐based distance calculation

A collection of k‐mers sampled at random from the nuclear genome encodes a remarkable amount of information. For a genome of size *n*, and ignoring repeats, a k‐mer of sufficient size (log_4_ *n*) will be unique in that genome with high probability. Helpfully, the probability of finding that k‐mer in another genome relates directly to the evolutionary distance to the other genome. Modelling two genome skims simply as sets of k‐mers *A* and *B*, we can define the fraction of shared k‐mers by the Jaccard index:

$$J=\frac{\left|A\cap B\right|}{\left|A\cup B\right|}.$$

*J* is intimately connected to the genomic distance *D* between the two organisms (Fan et al., 2015). Assuming all mutations to be equally likely, we can estimate *D* as.

$$D=1-{\left(\frac{2J}{1+J}\right)}^{\frac{1}{k}}.$$

Moreover, *J* can be computed efficiently by selecting as few as 10^3^ k‐mers from the set of all k‐mers using the min‐hashing technique (Ondov et al., 2016). However, the min‐hashing technique still needs to have high coverage of the genome, from which it then selects a subset of k‐mers. Thus, this method assumes the coverage is high enough that each k‐mer is sampled at least once in the original data (e.g. before selecting a subset of k‐mers). Recently, we developed a method called Skmer that allows for accurate estimation of genomic distance with extremely low (e.g. 0.1X) coverage, even when the coverage is unknown and in the presence of sequencing errors (Sarmashghi et al., 2019). Skmer uses k‐mer frequencies to estimate genome length, coverage, and sequencing error and uses the Jaccard index to compute genomic distance using a more complex version of the equation above. Because assembly is not needed, adding new species to the reference set of Skmer requires minimal preprocessing or indexing, and thus, is straightforward.

While Skmer has performed well in comparison to other assembly‐free methods (Sarmashghi et al., 2019; Zielezinski et al., 2019), our intention here is not to advocate Skmer specifically; our general arguments apply to other assembly‐free methods. Many such methods exist and they use a variety of signals to estimate distance. Other methods that use k‐mers include Mash (Ondov et al., 2016), Simka (Benoit et al., 2016), FFP‐based methods (Sims, Jun, Wu, & Kim, 2009) and AAF (Fan et al., 2015; Sarmashghi et al., 2019). Another family of existing methods use the length distribution of matched substrings to estimate the distance (e.g. Kr (Haubold, Pfaffelhuber, Domazet‐Loso, & Wiehe, 2009), spaced words (Leimeister, Boden, Horwege, Lindner, & Morgenstern, 2014) and kmacs (Leimeister & Morgenstern, 2014)). In particular, the SpaM family of methods (Lau, Dörrer, Leimeister, Bleidorn, & Morgenstern, 2019; Leimeister, Dencker, & Morgenstern, 2019) have been tested under conditions with low coverage with promising results. Yet, others, such as FastANI (Jain, Rodriguez‐R, Phillippy, Konstantinidis, & Aluru, 2018) and Co‐phylog (Yi & Jin, 2013), use micro‐alignments (see Zielezinski et al., 2019).

### 4.2 Sample identification

Once the genomic distance is measured, sample identification can follow the standard approach of finding the voucher species with the smallest distance to the query. While various alignment and assembly‐free methods can be used, we give an example using the tool Skmer, which has shown high accuracy in this setting. On data sets of *Anopheles* mosquitos, *Drosophila,* and birds with genome skims of size 0.1, 0.5, or 1 Gb (corresponding to ~0.5X–7X coverage), Skmer correctly identified the best match to every query skim. In a more challenging leave‐out analysis on the same data sets, we removed a query species and all of its closest matches (i.e. those, with distance lower than *x*% to the query for *x* set to 1, 2,…, 10) from the reference data set and asked whether the closest *remaining* match can still be identified; Skmer found the correct remaining match in every case for *Anopheles* and in 190 out of 210 and 375 out of 460 tests, respectively, for the *Drosophila* and bird data sets (Sarmashghi et al., 2019).

When an exact match to the query species is not available in the reference set, a phylogenetic approach is helpful. Phylogenetic placement can find the best placement of the query on a reference phylogeny of vouchers. Recently developed methods such as APPLES can perform phylogenetic placement using distances alone (Balaban, Sarmashghi, & Mirarab, 2020). Phylogenetic placement can improve accuracy of identification. For example, in a leave‐one‐out reanalysis of a data set of 61 lice genome skims (Boyd et al., 2017), APPLES was able to find the correct phylogenetic placement in 97% of cases, whereas simply picking the closest match was accurate in only 54% of the tests (Balaban et al., 2020).

### 4.3 Read cleanup and filtering

Before computing distances between DNA‐marks, several technical and conceptual issues must be addressed. Standard processing of reads, including adapter removal, deduplication and merging of paired‐end reads, is all needed and can be achieved using standard tools such as BBTools (Bushnell, 2014). A remaining type of preprocessing that is needed is dealing with extragenic DNA from sources other than the species of interest. While this is a serious issue, we note that it is not unique to a DNA‐mark approach, and rather represents an important challenge for the field, and we revisit it later in the article.

### 4.4 Why haven't genome‐wide approaches been adopted yet?

**FIGURE 3:**
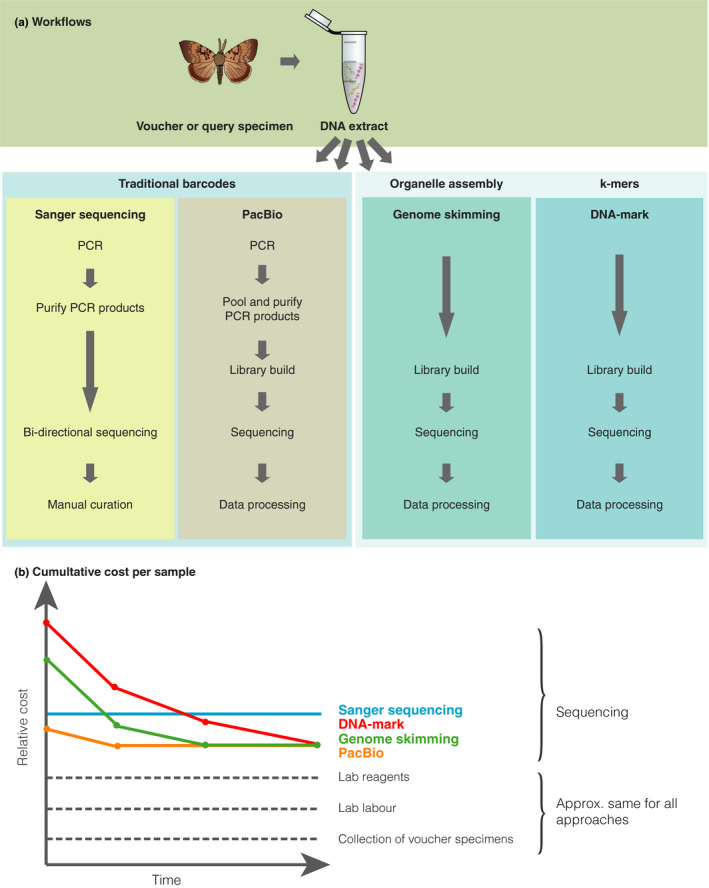
(a) Simplified description of the workflow process for generating different types of data that could be used for taxonomic identification. (b) Illustrative example showing that while the underlying cost of sample collection, vouchering and DNA extraction remain relatively constant with time as it is principally constrained by the cost of human labour, the cost of generating data using different next‐generation sequencing techniques is rapidly converging. Thus while, for example, the amount of shotgun sequence data needed to generate a species‐specific k‐mer profile is considerably more than is needed to mine an organellar genome, the economic cost of generating that much more sequence data is rapidly narrowing. We argue this supports the rationale for exploiting genome skims fully as a tool to complement traditional barcoding

One valid question is why such approaches have not already been adopted? First, until recently, shotgun sequencing costs per unit sequenced have simply been prohibitively expensive. Nevertheless, as sequencing costs per base continue to drop, the end‐to‐end costs will be increasingly dominated by processes necessary to the data generation (Figure 3). This includes, for example, the salaries of staff paid to collect voucher samples, extract and generate the DNA data, assemble and run QC on the results, and ultimately upload the data and accessory information into reference databases. Thus, while the difference purely in economic cost of PCR versus shotgun sequencing may at first look significant, the difference in true cost becomes minimal (Figure 3). Second, it might be assumed that the computational burden associated with any NGS‐based method is high. However, as already alluded to above, computational burdens for assembly‐free methods are considerably reduced. For example, the total running time (using 24 CPU cores) to compute 1,081 distances between all pairs of 48 avian genome skims using the Skmer tool took only 33 min (Sarmashghi et al., 2019). Other alignment‐free methods tend to be similarly fast. Third, while map‐free, alignment‐free methods of comparing genomes (including some based on k‐mers) have been known in the Bioinformatics community (Ondov et al., 2016), the power of k‐mer analysis for making inferences with low‐coverage genome skims was not well understood until recently, when a series of methods such as AAF, Skmer, Afann and *Read‐SpaM* were specifically designed to address this scenario (Fan et al., 2015; Lau et al., 2019; Sarmashghi et al., 2019; Tang et al., 2019) (Fan et al., 2015; Sarmashghi et al., 2019). Following these advances, user‐friendly software programs to efficiently use the k‐mer data are being actively developed, and new methods for improving their accuracy and usability are being designed.

We would argue that the only thing stopping this approach being implemented now is an exploration of its performance and potential, alongside the development of appropriate laboratory methods (such as efficient and cost‐effective library build protocols applicable to badly preserved voucher specimens, e.g. Troll et al. (2019)) and development of reference databases with suitable infrastructure.

### 4.5 Open methodological questions

As mentioned above, methods for computing genomic distance from genome skims and for phylogenetic analysis of those distances exist. Despite the progress, several unanswered methodological questions need to be further explored by the research community. Some of the questions are computational in nature, while others are related to laboratory techniques and the curation of comprehensive reference libraries. In the following section, we briefly discuss what some of these might be.

## 5 COMPUTATIONAL QUESTIONS

### 5.1 Coverage

A natural question is what depth of coverage will be needed for accurate sample identification. The answer is not straightforward and will depend on many factors, including genome length, sequencing errors introduced due to either postmortem DNA degradation (Lindahl, 1993; Pääbo, 1989) or library preparation enzyme and platform sequencing chemistry, and perhaps even the genomic architecture (e.g. the prevalence of repeats and polyploidy). The required depth of coverage is also a function of the genetic similarity between taxa. For example, the coverage required to distinguish a human from a chimpanzee sample would be higher than human from gibbon, simply as the former pair share many more k‐mers than the latter. Thus, a single number will not be universally applicable to different groups. Moreover, within‐species diversity is highly variable across the tree of life (Leffler et al., 2012). Nevertheless, our initial studies show that for species‐level identification, 1X coverage may be sufficient in most cases (Sarmashghi et al., 2019), and thus given our aforementioned argument that labour, not sequencing, is the bottleneck, perhaps, using a fixed sequencing effort (say, 2 Gb per species) would suffice in most cases. The required coverage is also a function of the method used for comparison. For example, the SpaM family of methods report higher accuracy than Skmer with low coverage for very large distances (Lau et al., 2019). Thus, more research is needed to characterize the exact resolution that can be obtained for a given coverage. Such research would entail simulation studies, empirical data and theoretical results that help us predict lower and upper bounds of distance that can be computed at a desired level of accuracy using specific methods and for different types of species with different genomic architectures (e.g. repeat structure).

### 5.2 Population‐level characterization

Related to the question of coverage is the question of resolution: Can a DNA‐mark distinguish groups at the subspecies level, thus both provide an identification tool at this resolution, and in doing so potentially complement, or even provide a relatively simple alternative to current population genomic tools used for population‐level assignment such as tools such as SNP typing assays (Wang et al., 1998), reduced representation library methods such as RADseq (Baird et al., 2008), GBS (Elshire et al., 2011) and the like, or even transcriptome and genome resequencing? Current methods such as Skmer tend to have very high accuracy for distances as low as 10^–2^ and reasonable accuracy for distances in the 10^–3^ range. For some groups, subspecies identification will require finer resolution. Accurately computing even lower distances despite low coverage (e.g. 1–5X) may be possible with improved methods. We believe increasing the resolution will require more complex modelling of the genomic structure, and in particular the profile of the repeated k‐mers across the genome. However, disentangling repeat structure from the k‐mer frequency profiles observed due to the random coverage of the genome is not easy and will require new algorithms.

### 5.3 Mutational models

Any measure of genomic distance is tightly linked with mutational processes that are modelled. Many of the existing k‐mer‐based methods (including Skmer) make simplifying assumptions about the evolutionary process, such as ignoring repeats and assuming a uniform distribution of mutations. These assumptions have been made mostly for methodological convenience. It is possible to relax many of them with further modelling. For example, uneven rates of evolution can be modelled using log‐det distances (Lockhart, Steel, Hendy, & Penny, 1994), and repeat structure can be estimated and accounted for in distance calculation. Future work should explore more advanced methods that relax many of the current assumptions.

Most k‐mer‐based methods directly model substitutions, but not processes such as insertions and deletions, gene duplications and losses, abundant repeats, polyploidy, and horizontal gene transfer. Some of these mutation types (e.g. short indels) also reduce the Jaccard index similarly, but not identically, to substitutions (a short indel, just like substitutions, reduces the Jaccard, but it can also slightly change the genomic length); thus, Jaccard‐based methods are expected to be robust to such events. Nevertheless, the robustness of the k‐mer‐based methods broadly and Jaccard‐based methods more specifically needs to be tested and improved in the face of complex mutations such as large‐scale duplications. This is especially important for plants and other organisms with complex genomic architecture. Moreover, the presence of complex mutations could itself be used as signal for detecting species and subspecies, but the challenge will lie in developing methods that can detect such differences between pairs of low‐coverage genome skims.

### 5.4 Sequencing technology

The exact choice of the sequencing technology will affect not only the lengths of sequences generated and sequencing error rates, but can also introduce biases through preferential sequencing of certain regions over others due to GC content, etc. (Browne et al., 2020). All of these may impact the accuracy of k‐mer‐based methods. In practice, it may also be that a reference data set would be composed of skims sequenced with different technologies. Would query searches against such databases remain unbiased? Since k‐mers break down long sequences into short ones anyway, there is reason to hope that they will remain robust to the choice of the sequencing technology. Nevertheless, empirical tests with mixed sequencing technologies currently do not exist.

### 5.5 Sampling

If reference databases are not comprehensive, and this goes for any reference database whether traditional barcode, organelle genome or k‐mer reference databases, taxonomic assignments of queries can suffer. Besides developing reference libraries with denser sampling, a phylogenetic perspective can also be helpful. In order to improve the characterization of samples, the metagenomics community has developed methods to both place a single sample in the phylogenetic context, and to compare multiple samples with each other (Brady & Salzberg, 2009; Janssen et al., 2018; Lozupone & Knight, 2005; Matsen, 2015; Matsen, Kodner, & Armbrust, 2010; Nguyen, Mirarab, Liu, Pop, & Warnow, 2014). Considering phylogenetic relationships between the query and reference sequences, we can look for the largest taxonomic level (e.g. a genus, family, or class) in which the query can be confidently placed. To this end, we have developed algorithms that combine k‐mer‐based distances with phylogeny‐based placement (Balaban et al., 2020). However, phylogenetic placement of genome skims can further benefit from methods that better characterize placement uncertainty, model rate variations and gene tree discordance across the genome, and incorporate complex substitution models.

### 5.6 Extragenic DNA

The most pernicious challenge is the possibility that the generated sequence data derives from more than one source. That is, voucher samples might not only contain DNA from the target species, but also that from other organisms. This could be from naturally impure voucher samples, for example endophytes associated with plants, or the gut contents of preserved insects, or even simply a result of microbial driven degradation. Alternatively, it could derive from contamination during the laboratory procedures, or even library bleeding during sequencing as has been reported for some Illumina platforms (Kircher, Sawyer, & Meyer, 2012; Sinha et al., 2017) and which may yield impure data sets. While conventional PCR or genome skimming approaches are not immune to contamination, identification and removal of contaminants is a much more straightforward process.

A recent study showed that for assembly‐free methods of genome matching, estimates of genomic distance are negatively impacted if contamination are not detected (Rachtman et al., 2020). Using both mathematical modelling and empirical data, the authors elucidated how the amount of contamination and the similarity of the contamination across skims being compared interact with negative impacts of contamination. Contaminating sequence reads can impact k‐mer‐based measures of distance in complex ways. The most damaging scenario is when both the query *and* the reference skims are impure, especially if the impurity of the query skim happens to be similar to that of some reference skims. In a scenario like that, the estimated distance from the query to a reference may be low, not because of the phylogenetic similarity but because of the similarity in contaminants. This can lead to underestimation of distances and, potentially, an incorrect identification.

One approach to deal with sample impurity is to filter out reads suspected to be contaminants. Existing methods such as BLAST or Kraken (Wood, Lu, & Langmead, 2019) can be used to search reads against databases of known contaminants. For example, if the sample is known to be of an insect, we can match reads against databases of bacteria, fungi, viruses and mammals. Any strong matches to these can be then eliminated. The analysis by Rachtman et al. (2020) has shown filtering using Kraken‐II to be effective in reducing the negative impacts of contamination, but only when the contaminants have relatively close matches to the contaminant reference library (e.g. a match with up to 5%–10% genomic distance). This observation leaves us with a methodological gap, namely efficient yet more effective methods of read matching at higher distances. These search methods should go beyond (near) exact matching to species available in the contaminant database, as those databases will always be incomplete. Instead, they should use the databases as a guide to broadly find reads that have likely originated from organisms other than the clade of interest. An alternative to this “exclusion‐filtering” method is inclusion‐filtering: designing methods that can identify reads that have, in fact, likely originated from some organism in the clade of interest.

### 5.7 Mixture analysis

The existing methodology for k‐mer‐based analysis of DNA‐marks mostly assumes the sample is of one target species (plus contaminants). Akin to metabarcoding, we can imagine a scenario where meta‐DNA‐marks are obtained from samples that include a mix of species of interest. For example, the sample may include a mix of several insects that are hard to physically separate. Or it may be bee‐bread, the collection of pollen from several plants and fungi that constitute the food source in a bee nest. A similar challenge is presented when the sampled genome is a recent hybrid of known species. Can a DNA‐mark from a mixed sample be decomposed into its constituent parts? While designing methods to solve this problem is not trivial, the success of the metagenomic field in developing methods for dealing with mixed samples makes us optimistic that methods for deconvoluting a DNA‐mark into their constituent species can be developed in the near future. As mixtures (and especially hybrids) of eukaryotic species are expected to consist of fewer species than bacterial species, we believe developing new methods specifically targeted at eukaryotic genome skims is a fruitful direction for future research.

## 6 SAMPLE COLLECTION, LABORATORY AND SEQUENCING DEVELOPMENTS

As mentioned above, the DNA‐mark approach could be complicated by sample impurity. Impurity can arise at all steps of the workflow, the very basal step of which is the point of sample collection. As with other approaches for DNA reference data generation, it is best to collect samples for DNA extraction and sequencing that contain as little DNA from other sources as possible, for instance avoiding obvious endophytes on plants and avoiding contamination by one's own DNA and from other sources during collection.

When generating all types of reference data, DNA‐mark reference data included, we need to do it efficiently, cost‐effectively and reliably and ensure that it causes minimal destruction to voucher specimens. For generation of DNA‐mark reference data, and to some extent all of this is valid for other approaches too, this can be achieved by following validated and standardized workflows and pipelines. Importantly, these should seek to (a) minimize (cross) contamination during laboratory work, through, for example, working in pre‐ and post‐PCR laboratories and in clean working environments, and by minimizing hands‐on‐labour, for example through semi‐automated laboratory processing on robots and semi‐automated bioinformatic pipelines, (b) simplify DNA extractions so they are pure and relatively universal across sample types, and (c) ensure that protocols for preparation of DNA extracts for sequencing, the so‐called library build, are as simple as possible, that they allow low quantities of input DNA, and that they account for potential artefacts such as “library bleeding,” which if not taken into account can cause false assignment of sequences to samples and thereby contaminate samples (Kircher et al., 2012; Sinha et al., 2017). With regard to sequencing platforms, these need to be cheap, high‐throughput, simple to use and reliable.

## 7 CONCLUDING REMARKS

A community effort will be needed if we are to effectively address the aforementioned challenges associated with using k‐mers in general, that is to (i) characterize the resolution that can be obtained for a given coverage for species with different genomic architectures, to (ii) investigate whether—and how—DNA‐marks can distinguish groups at the subspecies level, to (iii) test and improve the robustness of the k‐mer‐based methods in the face of complex mutations such as large‐scale duplications, to (iv) assess whether sample identification using k‐mers is robust across sequencing technologies, to (v) develop methods for phylogenetic placement of genome skims that allows a better characterization of placement uncertainty, to (vi) model rate variations and gene tree discordance across the genome and incorporate complex substitution models, to (vii) develop methods to allow for extragenic DNA to be filtered out even when contaminants have high distance matches to the contaminant reference library, and lastly, to (viii) develop methods for k‐mer‐based identification of several taxa within a sample. In parallel with these efforts, the required curated public DNA‐mark reference database against which queries can be run could be established. Such a reference database could, for example, be comprised of both the processed genome skim data and the assembled organellar genomes that can be mined from genome skims. This in turn would ideally be based on both data submitted by those deliberately aiming to contribute to the database, and mined from any pre‐existing publically available shotgun sequence data set—as long as sufficient controls are in place to ensure that such data are derived from the taxa it is labelled with (something that has plagued genetic studies, including those based on conventional barcoding, since the introduction of such databases (Mioduchowska, Czyż, Gołdyn, Kur, & Sell, 2018)). Given that such data would naturally complement well‐established initiatives such as those comprising of either barcode fragments such as the Barcode of Life Database (BOLD), and/or organellar and whole genomes such as in Norbol, PhyloAlps and DNAmark and the various initiatives under the Earth BioGenome Project, one desirable strategy might even be to simply embed the framework within one of these resources.

With such an initial framework in place, our hope is that this will provide both a valuable tool with which to complement conventional barcoding, and also open up new research questions (Table 1). Obvious potential avenues include exploring whether such approaches might also be used to identify the genetic sources within more complex DNA mixtures, as is currently done using DNA metabarcoding of, for example environmental DNA or DNA extracted from bulk specimen samples (Taberlet, Coissac, Pompanon, Brochmann, & Willerslev, 2012). Other potential avenues could be as a new tool for reconstructing phylogenies, analysing the genetics of populations and even identifying samples to the individual level.

**TABLE 1:** Overview of sample collection, laboratory and sequence processing steps and of applications of DNA‐based sample identification methods

	Traditional PCR‐based barcoding		Genome skimming^a^ using next‐generation sequencing		Earth BioGenome Project^b^
	Sanger sequencing	Next‐generation sequencing	Organelle assembly	k‐mers	Whole‐genome assembly
Sample collection					
Sampling efforts	Same	Same	Same	Same	Same
Voucher specimen	Same	Same	Same	Same	Same
Laboratory					
Extraction	Standard	Standard	Standard	Standard	High molecular weight
PCR of marker region	Yes	Yes	No	No	No
Library build	No	Yes	Yes	Yes	Yes Multiple types
Sequence read processing					
Initial trimming of sequence reads	Yes (manual)	Yes	Yes	Yes	Yes
Quality check of barcode sequence	Yes (manual)	Yes	Yes	No	Yes
Creating k‐mer profile	No	No	No	Yes	No
Assembly of organellar genome	No	No	Yes	Optional	Yes
Assembly of whole genomes	No	No	No	No	Yes
Applications					
Identification at taxonomic species level	Sometimes	Sometimes	Yes	Yes	Yes
Taxonomic identification of simple samples	Yes	Yes	Yes	Yes	Yes
Taxonomic reconstruction of complex samples	Yes	Yes	Yes unless contains very closely related taxa	Perhaps—remains to be fully explored	No
Population‐level resolution	Rarely—requires population structure and high genetic divergence between populations	Rarely—requires population structure and high genetic divergence between populations	Sometimes—if characterized by unique organelle haplotypes	Perhaps—to be fully explored	Yes if sufficient population structure exists
Discerning individual‐level information	No	No	No	Perhaps	Yes

^a^ Requires ca. 1 gbp of shotgun sequencing (Coissac et al., 2016).

^b^ If funding can be secured, the EBP aims to generate chromosome‐level genome assemblies for all known eukaryote species (Lewin et al., 2018).

## AUTHOR CONTRIBUTIONS

This opinion was conceived and cowritten equally by K.B., S.M., V.B. and M.T.P.G.
